# OMR-Arena: Automated Measurement and Stimulation System to Determine Mouse Visual Thresholds Based on Optomotor Responses

**DOI:** 10.1371/journal.pone.0078058

**Published:** 2013-11-15

**Authors:** Friedrich Kretschmer, Viola Kretschmer, Vincent P. Kunze, Jutta Kretzberg

**Affiliations:** 1 Retinal Circuit Development & Genetics Unit, Neurobiology Neurodegeneration & Repair Laboratory, National Eye Institute, National Institutes of Health, Bethesda, Maryland, United States of America; 2 Neurobiology, University of Oldenburg, Oldenburg, Germany; 3 Computational Neuroscience, University of Oldenburg, Oldenburg, Germany; 4 Research Center Neurosensory Science, University of Oldenburg, Oldenburg, Germany; Dalhousie University, Canada

## Abstract

Measurement of the optomotor response is a common way to determine thresholds of the visual system in animals. Particularly in mice, it is frequently used to characterize the visual performance of different genetically modified strains or to test the effect of various drugs on visual performance. Several methods have been developed to facilitate the presentation of stimuli using computer screens or projectors. Common methods are either based on the measurement of eye movement during optokinetic reflex behavior or rely on the measurement of head and/or body-movements during optomotor responses. Eye-movements can easily and objectively be quantified, but their measurement requires invasive fixation of the animals. Head movements can be observed in freely moving animals, but until now depended on the judgment of a human observer who reported the counted tracking movements of the animal during an experiment. In this study we present a novel measurement and stimulation system based on open source building plans and software. This system presents appropriate 360 stimuli while simultaneously video-tracking the animal's head-movements without fixation. The on-line determined head gaze is used to adjust the stimulus to the head position, as well as to automatically calculate visual acuity. Exemplary, we show that automatically measured visual response curves of mice match the results obtained by a human observer very well. The spatial acuity thresholds yielded by the automatic analysis are also consistent with the human observer approach and with published results. Hence, OMR-arena provides an affordable, convenient and objective way to measure mouse visual performance.

## Introduction

Since genetics has offered the opportunity to generate mice with specific modifications, they have become one of the standard laboratory animals in biological and biomedical research. Consequently, even though mice can hardly be considered particularly visually oriented mammals, they are frequently used to study function and diseases of the visual system. A whole arsenal of different behavioral tests has been developed to cover different aspects of rodent vision [1]. Some examples used for mice include the T-maze developed to study pattern discrimination [2], [3], the two-alternative-choice test [4] which can be used to determine visual acuity [5] and the Morris water maze [6] or the Barnes Maze [7] to study visuospatial learning. Another very common method to measure properties of the visual system such as contrast-thresholds, spectral sensitivities and spatial or temporal acuity, utilizes the optokinetic response (OKR) or the optomotor response (OMR). The measurement of these reflexes have been used for over fifty years to study the visual systems of different species (e.g. OKR [8]–[13] or OMR [9], [14] in fish, OKR [15] or OMR [16] in turtle, and OKR [17], [18] or OMR [19]–[23] in mouse). In particular in mice, optokinetic and optomotor reactions are used frequently to characterize differences in visual performance of different mouse strains [1], [17], [21], [24]. These reflexes are triggered when an animal (or human) visually perceives movement of large parts of the visual environment, e.g. when gazing at the landscape through the window of a moving train. The reflex behavior consists of distinct involuntary movements of body/head (OMR) or eyes (OKR), which stabilize the image of the visual environment on the retina. The contributions of these components in natural viewing behavior vary from species to species.

In experimental measurements of OKR and OMR behavior, typically a pattern of random dots [8], [18], a regular vertical stripe pattern [19], [21] or a sinusoidal grid [9], [20] is moved horizontally across the animal's field of view with a constant [20] or sinusoidally changing velocity [10]. While traditionally cylinders with painted or printed patterns were used for stimulation, digital stimulation techniques have significantly facilitated OKR measurements [17], [18], [25] by allowing fast and flexible adjustment of stimulus parameters.

One of the first setups that utilized a stimulation with computer monitors to measure OMR responses in mice was the *OptoMotry* system [20]. This popular, commercially available system can be considered as a standard to measure visual thresholds in mice that are used in many studies, e.g. [26]–[29]. Their system uses four computer monitors to present a virtual cylinder around the animal. The experimenter observes the animal from above and counts head/body movements in response to the stimulation. Additionally, the experimenter tracks the head of the animal manually with the computer mouse to readjust the position of the virtual cylinder. This readjustment guarantees that the mouse perceives a constant grating by maintaining a constant distance between the animal's head and the virtual cylinder when the mouse moves its head away from the center of the arena. This effect was previously ignored for physical cylinders with generally much larger diameters (typically 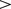
500 mm) and consequently smaller errors induced by head movements.

In contrast to the measurement of eye movements [18], [30], [31], the animal can move naturally in the *OptoMotry* system [20] and no fixation of the animal's head by using surgery to screw or glue the skull to a holder is required. However, a drawback of the system is that the quantification of tracking behavior is solely based on the judgment of a human observer during the experiment. The lack of an automated recording of the head/body position significantly restricts the possible analysis during or after an experiment. While double blind testing and multiple observers can improve the objectivity to some extent, an automated evaluation guarantees objective determination of visual thresholds and facilitates and speeds up such experiments even more.

For these reasons we developed a four-monitor based measurement and stimulation system that allows measurements based on the judgment of a human observer as well as a novel fully automated head tracking system suited for the objective measurement of visual thresholds. In this study, we show that the automatically determined spatial frequency response curve agrees very well with the results determined by a human observer and with previously published data. Our custom video-tracking algorithm is able to deal with continuously changing light conditions and determines the head-gaze of mice during experiments. All software and building plans for the OMR-arena will become available open source, allowing interested groups to replicate and use the system to measure visually evoked head and body movements in rodents.

## Methods

### Stimulation and recording hardware

**Figure 1:**
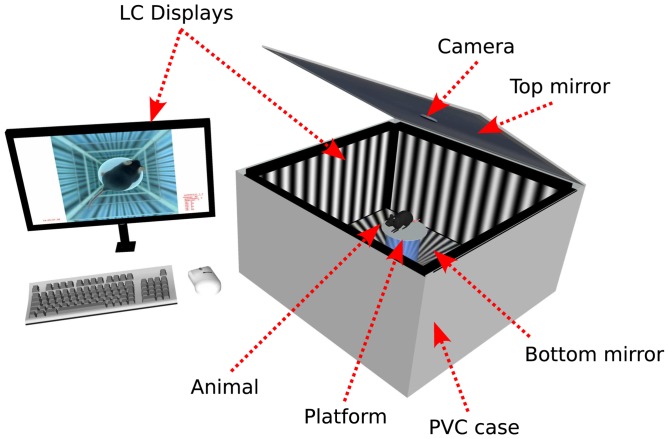
Overview of the setup. Four monitors are screwed into a PVC box. Top and bottom mirrors create the illusion of infinite profundity and hinder the animal from leaving the platform. A camera above the platform is used to monitor the inside of the arena. The stimulus parameters, the video recording of the behaving animal and the head-tracking can be observed by the experimenter on a fifth monitor.

The stimulus was presented on four 22″ LC displays (Nec Multisync EA222WMe) mounted to the inside of a custom built PVC box (690 mm
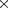
690 mm, height 354 mm), with a tightly closing lid, surrounding a centered, elevated circular platform (see figure 1). In accordance with previous observations [20] we also chose a diameter of 53 mm for the platform, which is large enough to allow the animal to safely stand, while being small enough to inhibit it from arbitrarily running around on it.

Two mirrors were attached to the floor and cover of the PVC box to create the optical illusion of an infinitely deep profundity, to prevent the animal from jumping off the platform. A glass mirror was used for the bottom floor of the PVC box to facilitate cleaning with water and 70% ethanol. Feces have to be removed frequently during the experiments, not only for hygienic reasons, but also because they disturb the reflection on the mirrors, causing the mice to jump off the platform more often. A PVC mirror was used for the ceiling to reduce the weight of the cover plate, which needs to be lifted for handling the animal in the arena.

The camera for the head-tracking (Logitech pro 9000) was integrated into this cover plate above the platform. The camera was removed from its casing to facilitate a precisely centered placement and to optionally remove the built-in infrared filter (to add an additional infrared light-source if it is required experimentally). The gray borders of the monitor around the actual display were covered with dull white adhesive foil to facilitate head-tracking by increasing the contrast between the animal's dark fur and the background. (In the case of measuring albino mice, by default, black foil should be used.)

The four monitors were connected to a Sapphire HD 4850 X2 graphics card through DVI. A fifth display was connected to the onboard graphics card of the PC mainboard (Phenom II, X6 with 2.81 GHz and 4 GB RAM) to control the experiments. The camera was attached to this PC via USB.

### Stimulation software

**Figure 2:**
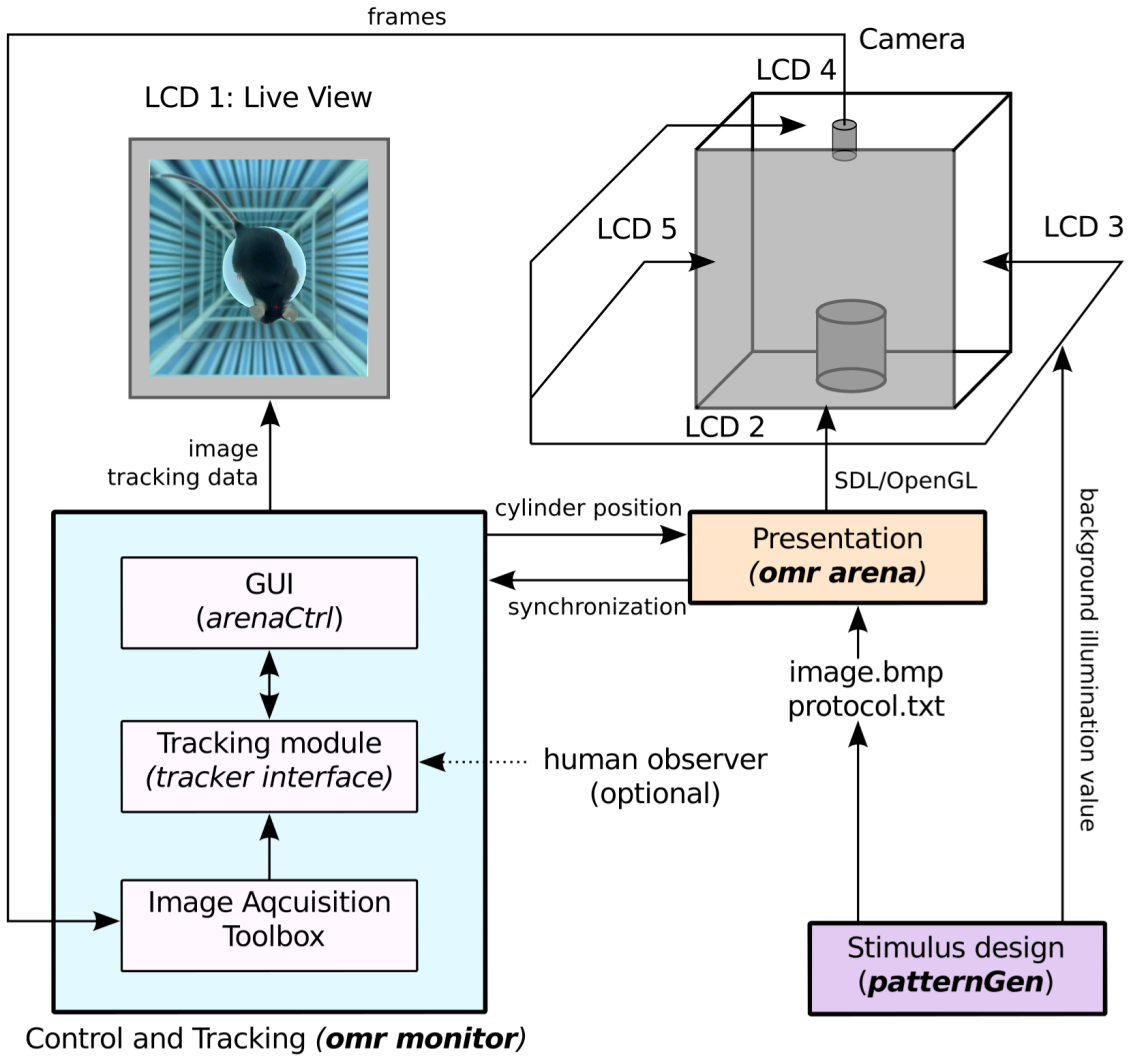
Schematic software overview. The program consists of three components. The component *patternGen* (purple) generates arbitrary stimuli, consisting of a texture (bitmap file) and a rotation protocol (text file). It also adjusts the background illumination value to gain a specific absolute intensity set by the user in the GUI. The *omr monitor* (light blue) implements the communication with the camera, the head tracking and the user interface during an experiment. Images are acquired through the MATLAB image acquisition toolbox and are used by the tracking module to determine the animal's head location. The live view is displayed in an on-screen GUI (*arenaCtrl*) in which the user can also adjust tracking parameters. The retrieved head position is used to recalculate the location of the stimulus pattern. The updated translation and rotation parameters are passed to the third component, *omr arena* (red), which presents the cylinder at the correct position on the four screens through SDL/OpenGL.

We implemented the stimulus software in C using SDL, a cross-platform multimedia library [32] and OpenGL [33] (see figure 2 for an overview). The software is able to present arbitrary stimuli on four displays simultaneously. (In contrast, the common MATLAB Psychophysics Toolbox [34], which we used in a previous version of the stimulation software [23], only fully supports two displays.)

A stimulation protocol and an image file are passed to the software component responsible for stimulation (*omr arena* in figure 2). The stimulus presentation component then draws a cylinder of an arbitrary radius on the four stimulation monitors using the image file as a texture and rotates the cylinder according to the stimulation protocol. While the program can run in stand-alone mode, an additional interface to MATLAB was implemented to facilitate control of the experiments and communication with the video tracking system. Further documentation will become available on our website (www.openetho.com).

### Stimulus design software

**Figure 3:**
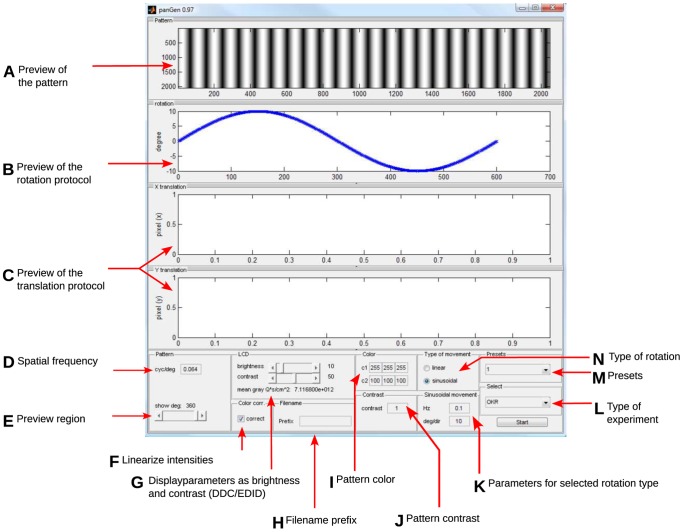
Stimulus generation tool. The graphical user interface of the *patternGen* component, including illustrations of (A) the stimulus pattern, (B) the rotation protocol, (C) the translation protocol (not used in OMR experiments). The user sets parameter values of (D) the desired spatial frequency in cyc/, (E) the range of the pattern displayed in the preview, (F) activation of linear color space (color values are mapped according to display calibration), (G) brightness and contrast of the stimulation displays, (H) a filename prefix for the experiment (optional), (I) color and (J) contrast of the pattern, (N) selection of linear or sinusoidal stimulus rotation, (K) parameters for the selected type of rotation (for linear movement: velocity, frequency and amplitude). Moreover, the user can load (M) a preset, consisting of previously saved settings, or (L) select a different experiment type (for future paradigms other than OMR/OKR).

To design the stimulus textures and the stimulus protocols, a MATLAB program (*patternGen* in figure 2, see figure 3 for a screenshot of the user interface) was implemented, allowing the modification of several stimulus parameters (e.g. spatial frequency, color, contrast and velocity of the moving pattern). Moreover, the program adjusts the stimulation to the specific properties of the LCD monitors used for stimulation. For our LC displays, measuring the spectral composition, as well as the absolute intensities at different gray levels (RGB values) and contrast settings of the displays with a calibrated spectrometer (USB-4000, Oceanoptics, Inc. Dunedin, FL, USA) revealed a non-linear increase of the intensity. The stimulus design tool reads the previously measured optoelectronic parameters of the stimulation monitors to correct contrasts and colors accordingly, generating a precisely linear in- and decrease of contrast. Moreover, the program sets the background illumination level of the screens to a specific quantum flow, to gain a well-defined mean illumination intensity within the experimental arena. The stimulus design software also defines the maximum (white) and minimum (black) light intensity. In our experiments, they were set to the values used in previous studies (mean black intensity 0.22 cd/m^2^ and mean white intensity 152.13 cd/m^2^, [20]).

### Tracking algorithm

Head-tracking serves two purposes. First, the data is used to automatically maintain the distance of the animal to the virtual cylinder and therefore, the perceived spatial frequency of the grating constant. Second, this data (gaze angle over time) is recorded and used to evaluate all experiments.

**Figure 4:**
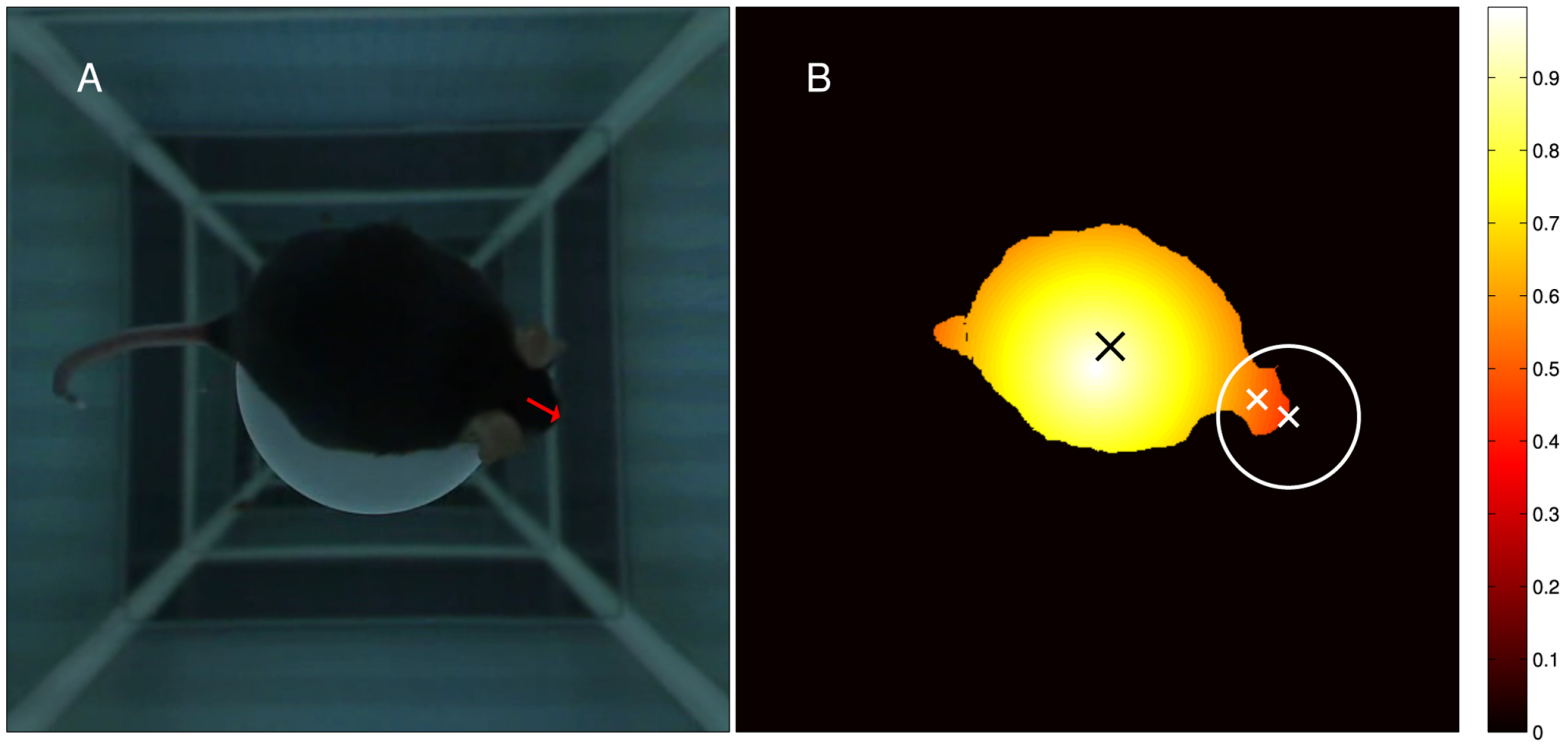
Head tracking. During experiments, the gaze of the freely moving animal (red arrow) is tracked continuously (A). Algorithmic steps to determine the gaze (B): Segmentation is based on a color-threshold, which is automatically determined by analyzing the pixels in the platform region. To facilitate the detection of the nose position, coordinates are weighted with a function ranging from 0 at the border of the region around the platform that the mouse can reach with its nose to 1 at the center of the platform. The center of gravity (black X) is calculated based on weighted pixels (see color bar). It is usually located in the animal's hind-quarters region. The nose (white X in circle) is usually detected as the pixel farthest from the center of gravity. The position between the ears (white X) is determined as a center of mass in a circular region (white circle) around the location of the nose. The head-gaze (red arrow in A) is calculated as the vector from the position between the ears to the position of the nose. See supporting material S1 for an example video visualizing head tracking.

A custom tracking algorithm was implemented in MATLAB, using the MATLAB Image Acquisition Toolbox to access the camera. The tracking algorithm follows the head gaze of the mouse while stimulating with arbitrary 360 patterns. Figure 4 depicts an example of a preprocessed image and the calculated head gaze.

Prior to analysis, a color threshold 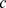 had to be found which reliably separated the dark fur of the mouse from the white platform (or in case of albino mice the white fur from the black platform.) For this purpose, the pixels representing the area of the platform in the first ten frames of the recording were used. We determined the subset of these pixels for which all three RGB-color values were 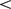
100. The RGB-color threshold 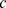 used for the analysis of all following frames was defined as the average RGB value of this subset of pixels.

For each of the subsequent frames the following algorithm (an improved version of [23]) was used:

Binarization and segmentation: Binary masking was applied to discard the area outside of an empirically determined circular region around the platform, which was impossible for the mouse to reach. The frames were then converted to a binary representation by applying the previously calculated color threshold 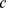. The image was segmented and everything except the largest object was discarded. We call the resulting set of all coordinates belonging to the mouse body 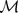.

Weighting of coordinates: To locate the nose position, all coordinates belonging to the mouse body were weighted. They were multiplied with a function 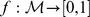 ranging from 0 at the border of the reachable circular region around the platform to 1 at the center of the platform. We call the weighted set of coordinates belonging to the mouse body 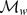, then
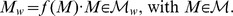



Center of gravity: In the next step, the center of gravity 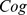 of the weighted coordinates of the object was calculated:
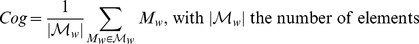
This coordinate usually corresponded to the hind-quarter region of the animal which is broader than its front, due to the size of the abdomen. This weighting further improved the probability to find a coordinate closer to the tail than to the nose.

Nose position: Typically the coordinate belonging to the mouse body with the largest distance from 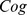 represents the nose position. The weighting helps to avoid the rare cases in which the calculated center of gravity is closer to the nose than to the tail. It makes sure that the probability of a pixel to be the nose position is higher the farther it is from the center of the platform. The nose position 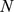 is determined as the weighted coordinate with the highest distance from the center of gravity 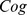:
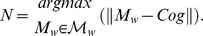



Head gaze: A circular masking was applied on the binary image around the location of the nose and another center of mass was calculated within this region. Due to the strong symmetry of the animal's head the identified position corresponds to a central location on the sagittal axis of the animal's skull. The head-gaze (see figure 4 and supporting video S1) is then calculated as the vector from the coordinate of the center of gravity of the head to the coordinate of the nose.

Additionally, the automated measurement system provides two different head-tracking algorithms, which were not used in this study. These rely on the tracking of artificial markers attached to the head of the animal. They perform tracking similarly well, but the attached marker can lead to distraction of the animal [23], [35].

### Feedback from head-tracking to stimulation

Since the animal moves freely on the platform, the head of the animal is only occasionally in the exact center of the arena. To achieve a constant spatial frequency of the perceived stimulus, it needs to be readjusted by shifting the center of the virtual arena to the head of the animal. In our setup, the position of the virtual cylinder is corrected continuously by setting the center of the arena to the head position determined by the on-line video tracking. Alternatively, the user can choose to use manual tracking similar to the *OptoMotry* system [20]. In this case, the center of the arena is set to the position where the user indicates with a computer mouse click. However, manual on-line tracking leads to larger errors in the estimated head position and longer feedback delays than automated tracking [23].

We noticed that the animals showed irritated behavior, similar to responses to looming stimuli, whenever the cylinder changed its position too quickly. Such situations sometimes resulted in fluctuating behavior of the system: a fast movement of the stimulus yielded a fast behavioral reaction of the mouse, which caused an additional movement of the cylinder. Therefore, an additional low-pass filter with a window length of 20 frames was applied to the previously described correction movement of the cylinder. This value was determined empirically by increasing the length of the filter gradually until no such fluctuations occurred.

### Automatic evaluation of tracking behavior

**Figure 5:**
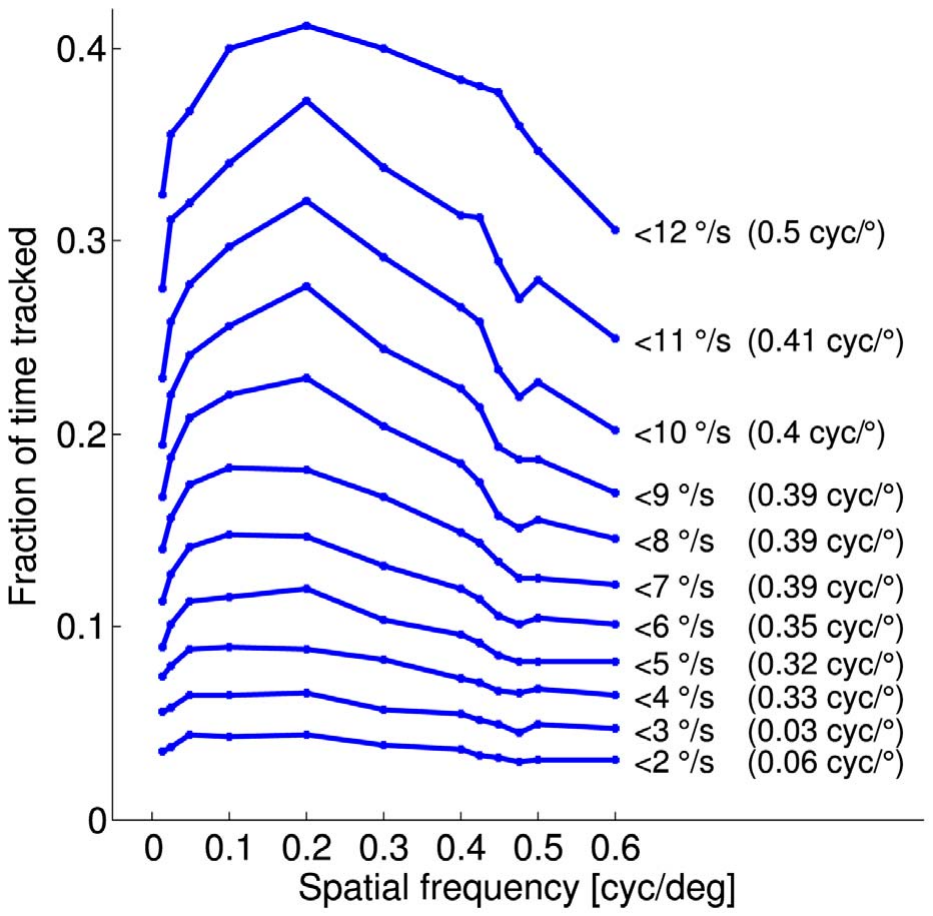
Effect of the variation of the discrimination criterion. The response curves were calculated on basis of the individual normalized responses of the 6 animals (as in figure 8) for different values of the discrimination criterion 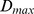 (values from 2 to 12/s, shown to the right of each curve). The fraction of time tracked (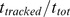) increases when softening the criterion, but the shape of the response curve as well as the estimated visual acuity based on logistic fits of the median responses (shown in brackets on the right) remains qualitatively similar in a certain range around the standard value of 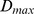 = 9/s.

To automatically detect and evaluate tracking behavior, the angular velocity of the head-movement was compared to stimulus velocity using the following algorithm:

Detect stimulus-related behavior: The sequences of the recorded head-angle and the stimulus positions were differentiated to retrieve the angular velocities 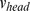 and 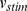, and then subtracted from each other. A frame was detected as stimulus-related behavior (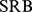), when the resulting difference was below the value of a discrimination parameter 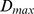:
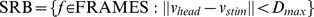
We used 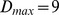 /sec as standard value for the maximum allowed velocity difference and analyzed the effect of varying this parameter (see figure 5).

To calculate the chance level, head movements recorded during the presentation of a static stripe pattern were compared to the stimulus velocity trace used in the other experiments. In this way, we quantified the amount of randomly occurring head movements that were classified by our method as stimulus-related behavior. For each animal, we determined the median of the randomly occurring SRB over 10 trials. The median of the six obtained median values was defined as chance level and set to zero by subtracting this value from all SRB values.

Quantify overall tracking performance: The overall tracking performance was then quantified as the ratio of the number of frames identified as stimulus-related behavior to the total number of frames.
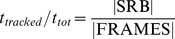



**Figure 6:**
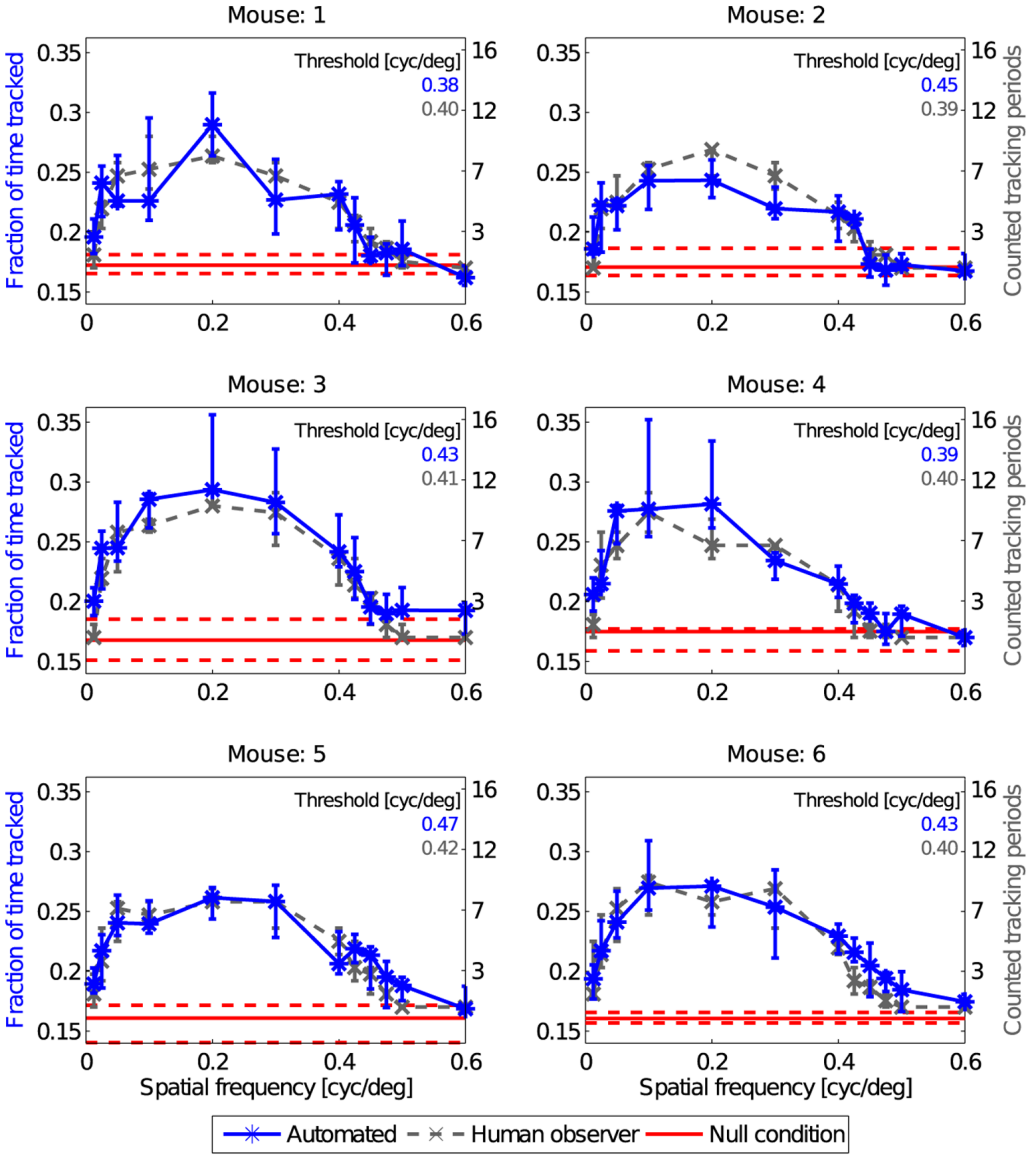
Individual OMR performances. The amount of tracking movements counted by the human observer (dashed gray line, not normalized, right axes) compared to the automatically determined response curve when using a discrimination criterion of 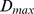 = 9/s (blue line, not normalized, left axes) for the six individual animals. All values are unnormalized medians over 10 stimulus presentations. The errorbars represent the variation of individual responses by showing the upper (75%) and lower (25%) quartiles of responses to each spatial frequency. The solid red line is the median for the measurements at null condition (not moving stimulus), the red dashed red lines represent the quartiles at this condition. The calculated individual spatial acuity thresholds are shown in the upper right corner of each graph.

**Figure 7:**
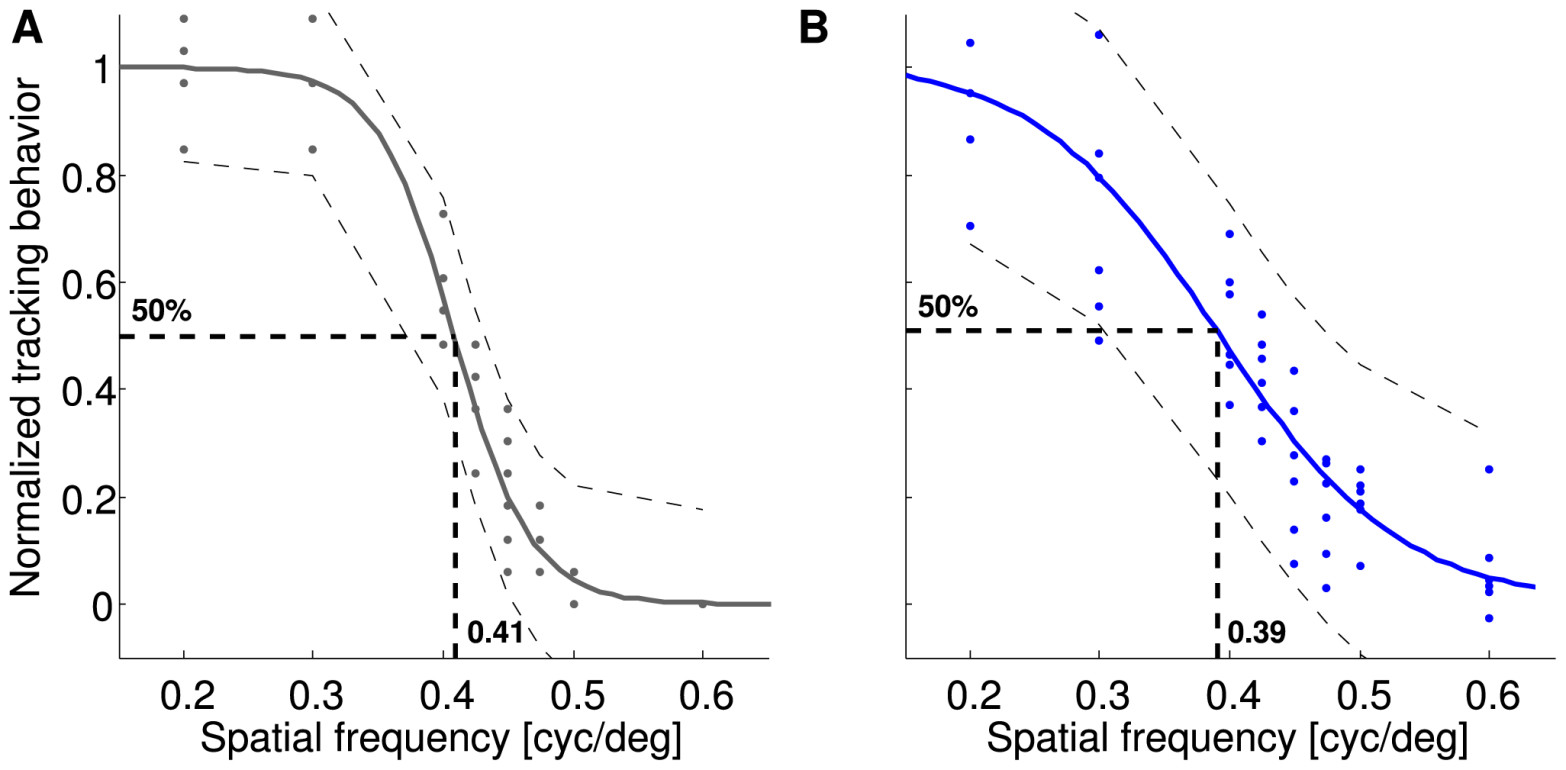
Determination of spatial frequency threshold. The descending slope of the response curves obtained by the human observer (gray dots, A) and of the automated analysis (blue dots, B) was fitted by a logistic function (gray and blue lines). Curve fitting was done based on the median values obtained for the six animals and each spatial frequency. Dashed lines indicate the prediction bounds, in which 95% of new measurements are expected to lie. The visual threshold was defined as the spatial frequency value at the inflection point (50% of the maximum response), resulting in 0.41 cyc/ for the human observer approach and 0.39 cyc/ for the automated analysis.

Determine the visual threshold: The responses at spatial frequencies above 0.2 cyc/ were fitted by a logistic function, using the nonlinear least absolute residual method (Matlab Curvefitting Toolbox 2.0, The MathWorks Inc., USA). The logistic function
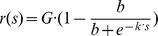
yields the behavioral response 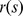 for the spatial frequency 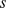. The parameter 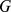 defines the maximum of the response. The parameter 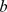 shifts the curve along the x-axis, 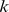 influences the steepness of the curve in its inflection point. 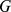, 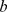 and 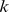 were free parameters in the fitting procedure, typical values were e.g. 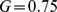, 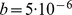, 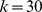. Fitting was done either based on data of individual animals (figure 6) or based on the median values obtained for each of the six mice to show the typical response (figures 7 and 5). In the later case median responses of all animals were normalized by setting the median of the medians obtained for the optimum spatial frequency to one.

We defined the spatial frequency eliciting half maximal responses (corresponding to the inflection point of the logistic function) as visual threshold, separating reliable tracking behavior from random agreement between stimulus and head movement. In principle, a less conservative choice of visual threshold would be possible, e.g. 25% of maximum response.

### Licensing

Whereas the stimulation software is released under the GNU General Public License (GPLv3) the stimulus generation requires MATLAB/Octave. The tracking modules additionally require the Image Acquisition toolbox and are hence released under GNU Lesser General Public License (LGPLv3). Due to the modular software architecture, a future version of the tracking algorithms could be implemented without the need to change any of the other components.

### Experiments

Six male, two month old, C57Bl/6J mice were kept in plexiglas cages (46
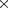
26
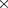
16 cm [L
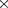
W
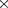
H]) and housed at room temperature and a 12 hr light/dark cycle. Food and water was available ad libitum. The mice were ear punched to recognize individuals. Experiments were conducted between 8 a.m. and 11 a.m. over a period of six weeks.

Stimuli consisted of a sinusoidal grating on which “stripes” gradually change from black (RGB [0 0 0]) to white (RGB [255 255 255]). Each experiment was composed of several measurements at different spatial frequencies (0.0125, 0.0250, 0.050, 0.1, 0.2, 0.3, 0.4, 0.425, 0.45, 0.475, 0.5 and 0.6 cyc/, corresponding to 4.5, 9, 18, 36, 72, 108, 144, 153, 162, 171, 180 and 216 pattern repetitions/360). Each spatial frequency was presented for 60 s, followed by a short break of approximately 20 s, in which the monitors showed a gray screen at medium light intensity. The stimulus pattern was rotated with 12/s around the animal, changing its direction every 6 s, resulting in a maximum of ten tracking movements per stimulus presentation. Each mouse was tested ten times with each spatial frequency. To account for the initial habituation and a possible decay in performance towards the end of an experiment, spatial frequencies were presented in a randomized sequence.

When using appropriate spatial frequencies, this stimulus amplitude and velocity evoked clearly visible head movements and yielded stable responses. In most cases, a mouse followed a single stimulus period continuously with its head without interruption by saccadic movements.

The behavioral responses to all spatial frequencies were video recorded and quantified both manually by the experimenter and automatically. Additionally, head movements were recorded during absence of motion to obtain the chance level for the automated analysis.

## Results

### Optomotor response curves

**Figure 8:**
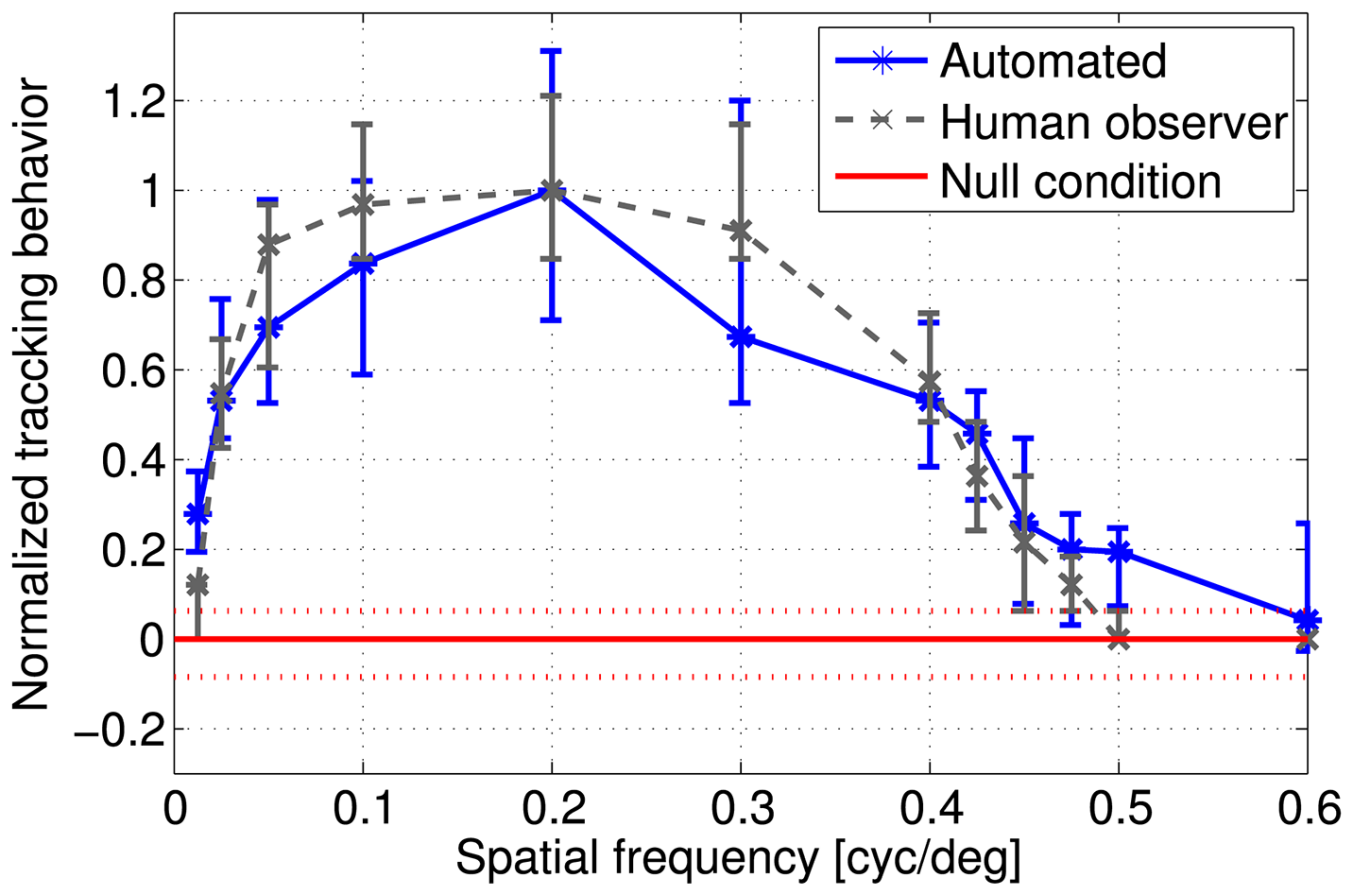
Automated and human observer analysis of OMR performance. The normalized response curves determined by the human observer (dashed gray line) and by the automated analysis (blue line, discrimination criterion 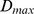 = 9/s) are similar. For each spatial frequency, the median of response of all mice was calculated based on the median values for each individual mouse. Responses are normalized by setting the median value obtained for the optimal spatial frequency to one. For the automated analysis, chance level was calculated as the median of the number of head movements matching the discrimination criterion by chance at null condition during absence of movement. It was set to zero (red line) by subtracting this chance value from all values obtained by the automated analysis. Errorbars and dotted red lines represent the variation between animals by showing the smallest and highest medians obtained for the six animals during stimulation and null condition. (See Methods for details).

The spatial frequency of a sinusoidal pattern moving with constant velocity strongly influences the optomotor response behavior of mice. This result was obtained by both a human observer as well as by automated evaluation (figure 8).

The human observer counted tracking movements on-line while the animals performed optomotor behavior. At too low spatial frequencies (0.0125 cyc/), the number of manually counted responses is close to zero (median 1 response, corresponding to a value of 0.12 when normalized to the maximum, see figure 8). For higher frequencies the amount of tracking behavior increased, reaching a maximum number of responses at 0.2 cyc/. The amount of tracking movements then decreased again to very low values at 0.45 cyc/ (normalized: median 0.21).

The automated evaluation compared the velocities of head and stimulus off-line, applying a criterion of a maximum difference of 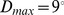/s to determine stimulus-related behavior. Behavioral performance was measured as the fraction of time 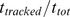, during which this criterion was met. Generally the response curve obtained by the automated evaluation matched the human observation (figure 8). Both evaluations yielded the maximum response at a spatial frequency of 0.2 cyc/ and a decreasing amount of stimulus related behavior towards both lower and higher spatial frequencies. In contrast to the manual evaluation, the automated analysis additionally allows for a comparison of responses to chance level. We defined chance level as the responses to a null condition when the stimulus pattern was not moving. Even under this condition, the animals performed by chance a low amount of head movements corresponding to the stimulus velocity used in the other experimental trials. This chance level (red line in figure 8) was subtracted from all automated analysis measurements. At very high (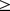
0.6 cyc/) spatial frequencies the amount of automatically classified tracking behavior was close to the responses at a null condition.

**Figure 9:**
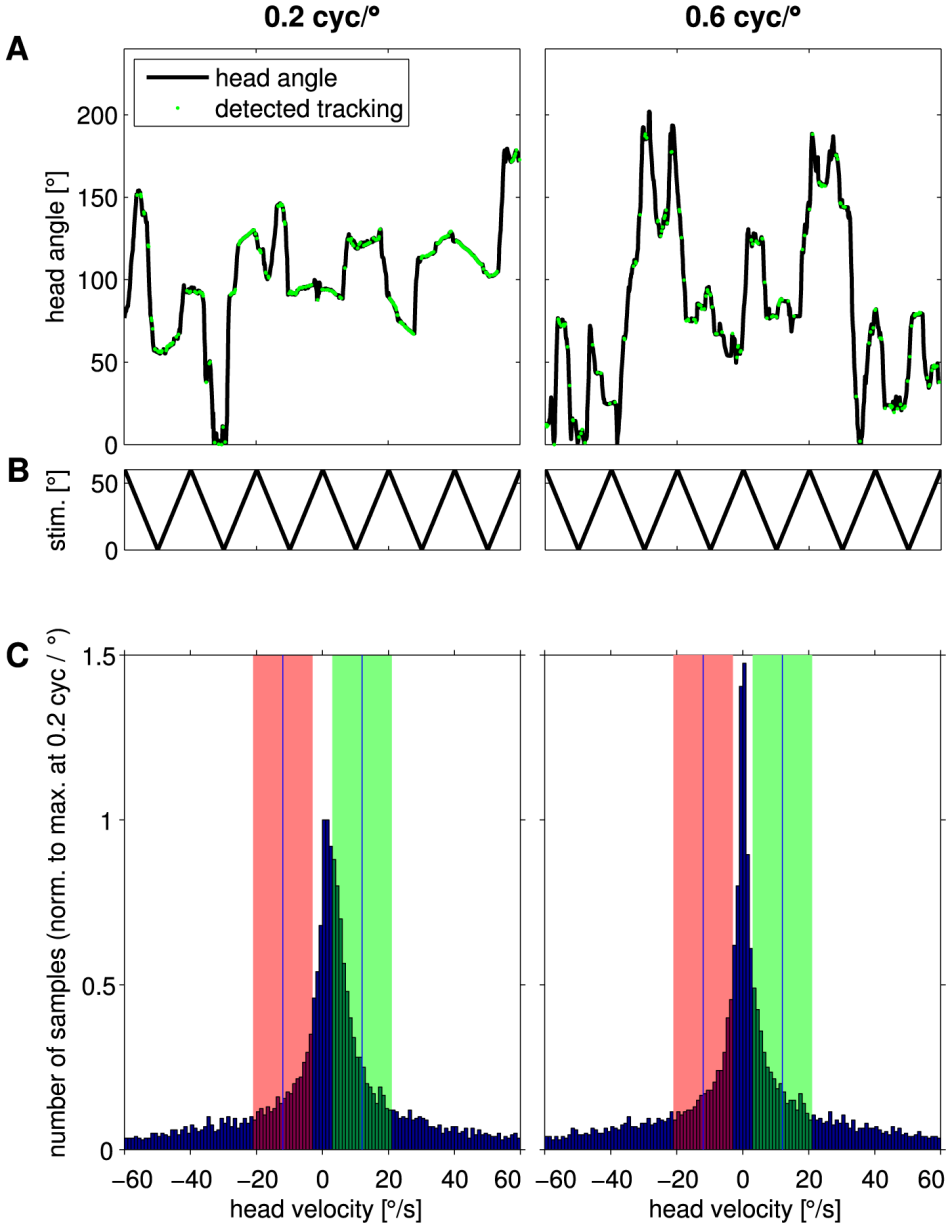
Recorded head-angles. A: Example course of the recorded head-angle over time of one animal at the optimal spatial frequency of 0.2 cyc/ (A left) and a spatial frequency of 0.6 cyc/ (A right), at which the human observer does not detect OMR behavior. Samples where the head angular velocity deviates less than 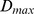 = 9/s from the stimulus velocity of 12/s are highlighted in green. Significantly more frames fulfill this criterion and are hence automatically detected as stimulus tracking behavior at 0.2 cyc/ than at 0.6 cyc/. B: Movement of the grating over time. C: Histogram of head velocities of all animals induced by the gratings with spatial frequencies of 0.2 cyc/ (left) and 0.6 cyc/ (right), both moving with 12/s. Positive numbers correspond to head-movements in the same direction as the stimulus moved, negative numbers to head-movements to the opposite direction. The green area indicates the region of head velocities that was tolerated as tracking behavior. To visualize the more strongly skewed distribution found for responses to 0.2 cyc/ in contrast to 0.6 cyc/, head-movements that occurred in the range of tolerated speeds but in the wrong direction are highlighted by the red area.

To clarify how the fraction of tracking time was calculated to measure behavioral performance, typical courses of the head angle of one mouse are shown in figure 9. The behavioral responses were elicited by the movement of a pattern with the optimal spatial frequency of 0.2 cyc/ (figure 9, left), or with a very high spatial frequency of 0.6 cyc/ (figure 9, right), which the mouse was apparently unable to perceive. In particular, for high spatial frequencies, it is hardly possible to determine by visual inspection of the recorded traces whether OMR tracking behavior occurred in response to the stimulus. However, the automated evaluation detected all frames which fulfilled the criterion for tracking movements (highlighted green in figure 9 A). In this study, we used a maximum deviation of 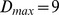 /s of the head velocity from the stimulus velocity. Hence, since the stimulus moved constantly with 12/s either to the left or to the right (figure 9 B), head movements in the same direction with velocities between 3 and 21/s were classified as tracking behavior. For stimulation with the optimal spatial frequency, the distribution of head velocities is skewed markedly towards movement in the correct direction, while it is symmetrical when a very high spatial frequency is used (compare red and green highlighting in figure 9 C). Moreover, the tolerated range of head velocities in the correct direction (highlighted in green) occurred much more frequently in response to optimal stimulation. When the animal was unable to perceive the stimulus, it generally moved less, leading to the high amount of slow head movements shown in 9 C (right). However, some short periods of fast head movements in the stimulus direction occurred incidentally in the case of too high (e.g. 0.6 cyc/) or too low (e.g. 0.0125 cyc/) spatial frequencies, or even in the absence of motion (null condition). These were classified by the algorithm as stimulus-related behavior (green dots in figure 9 A, right), even though they occurred by chance. Therefore, we subtracted chance level from all measurements to make sure that only stimulus induced behavior was considered for further analysis. The amount of time during which the mouse head movements match the stimulus movements was found to be higher the better the animal reacted to the stimulus.

### Visual acuity

Visual acuity has been classically defined as the highest spatial frequency eliciting a response that can be detected by the human observer [20]. Applying this criterion to our full data set resulted in a visual acuity of 0.425 cyc/. To introduce an objective measure that can be used for automated analysis, we fitted the sigmoidal decline of the response curve above 0.2 cyc/ with a logistic function (figure 7). We defined the visual acuity as the frequency at the inflection point of the logistic function (figure 8), eliciting 50% of the response maximum. Based on the judgment of the human observer, this threshold was reached at a spatial frequency of 0.41 cyc/ (figure 7 A). The automated analysis yielded a visual acuity of 0.39 cyc/ (figure 7 B).

Manual and automated analyses also led to very similar results when analyzing the behavior of individual animals. Both types of analyses showed the same dependency of tracking behavior on spatial frequencies and revealed similar thresholds for all mice (figure 6). When comparing the individual performances of the six mice tested in this study, all animals behaved similarly. Even the absolute values of the tracked fraction of time 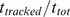 varied only slightly among the animals, e.g. at a spatial frequency of 0.2 cyc/ between median values of 0.24 (mouse 2) and 0.29 (mouse 3).

### Effect of the discrimination criterion

To test how the choice of the criterion to discriminate stimulus-correlated head movements influences the obtained response curves, the parameter 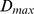 was varied (figure 5). We found that low values of 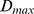 led to flatter response curves, until the responses for individual spatial frequencies became indistinguishable from each other (e.g. at 2/s). Meanwhile, high values of 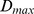 led to higher chance levels. For example in the extreme case of 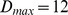 /s, approximately one third of the time was classified as stimulus-dependent behavior even for a spatial frequency of 0.6 cyc/, which did not trigger optomotor responses visible for a human observer. The reason for these high values for the fraction of time tracked, is the acceptance of very slow movements and even the absence of movement as tracking behavior. Since a stimulus velocity of 12/s values 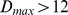 /s would classify movements even in the wrong direction as tracking behavior, they were excluded from our analysis.

Finally, visual acuity can be calculated more acurately for steeper response curves. Very high and very low values of 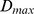 can lead to arbitrary estimates of visual acuity. We chose 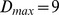 /s as standard value of the discrimination criterion (green regions in 9C) as the discrimination criterion that leads to a conservative estimate of visual acuity, avoiding false positive detection of seemingly stimulus-induced head movements. This parameter value provides a good compromise, combining a strong dependency of the time tracked on the spatial frequency and a relatively low chance level. However, since the qualitative shape of the response curve and the resulting estimate of the visual acuity remained similar within a certain range of values around 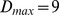 /s, the choice of the criterion is not critical.

## Discussion

### Comparison with existing methodologies

In this study we introduce the first fully automated system to stimulate, record and quantify visually induced head movements in a freely behaving rodent. Existing methodologies to measure visual thresholds in mice either rely on human observations of head movements (e.g. [19]–[21]) or on the recording of eye movements e.g. [17], [18], [36]), requiring an invasive fixation of the animal. Currently, the only systems that combine the measurement of head and eye movements in rodents shows striking differences in eye movements of freely moving and head-restrained rats [37]. Since this system has not yet been used to measure OMR/OKR behavior, the relative contribution of head and eye movements for this behavior are currently unknown for rodents. However, visual acuity measurements based on mouse OMR head movements (e.g. [20], [21], [38], also our study) lead to only slightly lower acuity estimates than studies using OKR eye movements in animals with fixated heads (e.g. [17], [39], [40]) or visual discrimination tasks (e.g. [5], [38], [41]). Similar results (typically in the range of 0.4–0.5 cyc/) obtained by fundamentally different approaches suggest that visual acuity can be determined reliably by either of them.

The presented open source measurement and analysis system enables an automated determination of visual acuity threshold measurements based on head movements in mice. During the behavioral experiments OMR stimuli are presented on multiple monitors. At the same time, the center of the head as well as the gaze direction of the animal is continuously tracked automatically, serving two purposes. First, head tracking is used to readjust the stimulus according to the position of the animal. This adjustment is important to maintain a constant spatial frequency during an experiment [20]. In a preceding study, the automatic readjustment was shown to work more precisely than the manual one performed by a human observer tracking the animal on-line with a computer mouse [23]. Second, the gaze angle is recorded for subsequent analyses. The automated data analysis identifies OMR behavior based on the difference between the recorded angular velocity of the animal's head and the stimulus velocity. The visual acuity is calculated as the inflection point of a logistic fit to the response curve.

In this paper, we show that the automated measurement and analysis system reveals very similar results to the human observer approach. The human observer counts periods of stimulus movement that elicited optomotor tracking head movements. The automated analysis determines the fraction of time, during which tracking head movements were detected. When normalized to their respective maxima, the resulting response curves agree very well for all individual subjects (figure 6) and also for the population median (figure 8). Therefore, both measurements can be considered to be equivalent.

The visual acuity determined in this study match published measurements well. The classical criterion of the highest spatial frequency still eliciting a response that can be detected by the human observer leads to a value of 0.425 cyc/. Our method to automatically determine the visual acuity based on the 50% value of a logistic fit to the response curve leads to slightly more conservative estimation. We calculated thresholds of 0.39 cyc/ for the automated analysis and 0.41 cyc/ based on the manually counted responses. These values are in very good accordance with several studies using a range of different methods to determine the visual acuity in mice [5], [20], [21], [41], [42].

A potential underestimation of the visual acuity might result from the use of LC displays for stimulus presentation. The mouse retina shows a particular opsin expression gradient with more green-sensitive M-cones in the dorsal and more UV-sensitive S-cones in the ventral retina [43], [44]. Hence LCDs cannot optimally stimulate the mouse retina due to the lack of UV light of the display's backlight.

It should be noted, that we designed the algorithm and chose the parameter to carefully avoid false positive detection of seemingly stimulus-induced movement. This choice results in a very conservative estimation of visual acuity. It would also be possible to use, for example, a threshold of 25% of the maximum head movements instead of the 50% inflection point as criterion to determine visual acuity, which would lead to thresholds of 0.47 cyc/ for the automated analysis and 0.44 cyc/ based on the manually counted responses. Since the automatically detected fraction of tracking behavior elicited by this spatial frequency still exceeds chance level considerably, this less conservative choice of threshold would still be legitimate and could be able to reveal visual acuity values almost as high as measured with behavioral discrimination tasks [38].

The automated analysis of recorded head-movements requires only one free parameter (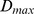) to determine an acuity response curve. We showed that the absolute value of this parameter is not critical, but that qualitatively similar results can be obtained when varying 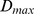 within a certain range around its standard value. It should be noted that the approach to discriminate tracking movements from other behavior based on the difference between head and stimulus velocities, is not equivalent to calculating the response gain. The gain, which is the ratio of tracking velocity to stimulus velocity, is a common measure to quantify tracking behavior based on eye movements in fixated mice [18] and has also been used to quantify the quality of tracking behavior based on head movements during optomotor stimulation in other species [45]. The analysis of the response gain during individual tracking periods might be a rewarding extension to be included in future versions of our measurement and analysis system.

### Practical advantages

A main practical advantage of the automated measurement and analysis system is that performing the experiments requires less expertise of the experimenter. In particular, the animals often use head and body tracking movements of very low amplitudes near their visual threshold. These can be observed only by well-trained personnel, but they are reliably detected by our algorithm. Moreover, handling of animals is easier than in other systems. Our method is less labor intensive and requires neither fixation of the animals nor attachment of artificial markers to their fur or skin.

The construction plans and software of the described setup will become available online under an open source license in the near future on the website www.openetho.com. The system requires only affordable and off-the-shelf hardware like the Logitech pro 9000 camera, a regular PC and LCD monitors, summing up to total costs of less than $3000.

The software is more flexible than commercial systems, providing the means to easily design and use arbitrary stimuli and cylinder movements. The MATLAB stimulus generation routine offers the choice of a variety of commonly used patterns (stripes, sinusoidal gratings, dots) and commonly used movements (linear/sinusoidal, clockwise/counterclockwise) and could easily be extended.

### Outlook

In the future, the measurement and stimulation system presented in this study could be automated even further with the goal to provide completely automated determination of visual thresholds. While in this study the recorded data was analyzed off-line after the experiment, on-line analyses could easily be included into future versions of the system. They could be used to control the experiment, either by providing a direct feedback to the experimenter on how the animal performed during the last trial, or by varying the stimulus automatically depending on the behavioral performance. In this case, the visual threshold could be determined in a fully automated way. Moreover, the closed-loop approach allows a direct change of the stimulus according to the animal's head position. Hence, it could also be useful for several other experiments and is not bound to OMR/OKR. We hope that users contribute their own ideas and routines to this project through our forum or the code repository (www.openetho.com).

In summary, we hope that the proposed open-source automated measurement and analysis system will help other scientists to measure visual thresholds of mouse-lines in an affordable, convenient and objective way. Our vision for future versions of the system is to generate one protocol that fully automates measurements for all relevant parameters of the visual system that an experimenter is interested in.

### Ethics statement

All animal experiments were performed in compliance with the guidelines for the welfare of experimental issued by the European Communities Council Directive of 24 November 1986 (86/609/EEC) and the laws of the Federal Government of Germany (Tierschutzgesetz; BGBl. I S. 1206, 1313 and BGBl. I S. 1934). Institutional approval for this study was obtained by Lower Saxony State Office for Consumer Protection and Food Safety (Az. 33.9-42502-04-07/1323).

## Supporting Information

Video S1 **Visualization of the tracking algorithm.** A video of one mouse placed on the platform in the setup performing OMR. To facilitate the detection of the nose position, coordinates are weighted with a function ranging from 0 at the border of the region around the platform that the mouse can reach with its nose to 1 at the center of the platform. The center of gravity (black X) is calculated based on weighted pixels (see color bar). It is usually located in the animal's hind-quarters region. The nose (white X in circle) is usually detected as the pixel farthest from the center of gravity. The position between the ears (white X) is determined as a center of mass in a circular region (white circle) around the location of the nose. The head-gaze (red arrow in A) is calculated as the vector from the position between the ears to the position of the nose.
(OGV)
